# 3D CNN-Based Speech Emotion Recognition Using K-Means Clustering and Spectrograms

**DOI:** 10.3390/e21050479

**Published:** 2019-05-08

**Authors:** Noushin Hajarolasvadi, Hasan Demirel

**Affiliations:** Department of Electrical and Electronics Engineering, Eastern Mediterranean University, 99628 Gazimagusa, North Cyprus, via Mersin 10, Turkey

**Keywords:** speech emotion recognition, 3D convolutional neural networks, deep learning, k-means clustering, spectrograms

## Abstract

Detecting human intentions and emotions helps improve human–robot interactions. Emotion recognition has been a challenging research direction in the past decade. This paper proposes an emotion recognition system based on analysis of speech signals. Firstly, we split each speech signal into overlapping frames of the same length. Next, we extract an 88-dimensional vector of audio features including Mel Frequency Cepstral Coefficients (MFCC), pitch, and intensity for each of the respective frames. In parallel, the spectrogram of each frame is generated. In the final preprocessing step, by applying *k*-means clustering on the extracted features of all frames of each audio signal, we select *k* most discriminant frames, namely keyframes, to summarize the speech signal. Then, the sequence of the corresponding spectrograms of keyframes is encapsulated in a 3D tensor. These tensors are used to train and test a 3D Convolutional Neural network using a 10-fold cross-validation approach. The proposed 3D CNN has two convolutional layers and one fully connected layer. Experiments are conducted on the Surrey Audio-Visual Expressed Emotion (SAVEE), Ryerson Multimedia Laboratory (RML), and eNTERFACE’05 databases. The results are superior to the state-of-the-art methods reported in the literature.

## 1. Introduction

Designing an accurate automatic emotion recognition (ER) system is crucial and beneficial to the development of many applications such as human–computer interactive (HCI) applications [1], computer-aided diagnosis systems, or deceit-analyzing systems. Three main models are in use for this purpose, namely acoustic, visual, and gestural. While a considerable amount of research and progress is dedicated to the visual model [2,3,4,5], speech as one of the most natural ways of communication among human beings is neglected unintentionally. Speech emotion recognition (SER) is useful for addressing HCI problems provided that it can overcome challenges such as understanding the true emotional state behind spoken words. In this context, SER can be used to improve human–machine interaction by interpreting human speech.

SER refers to the field of extracting semantics from speech signals. Applications such as pain and lie detection, computer-based tutorial systems, and movie or music recommendation systems that rely on the emotional state of the user can benefit from such an automatic system. In fact, the main goal of SER is to detect discriminative features of a speaker’s voice in different emotional situations.

Generally, a SER system extracts features of voice signal to predict the associated emotion using a classifier. A SER system needs to be robust to speaking rate and speaking style of the speaker. It means particular features such as age, gender, and culture differences should not affect the performance of the SER system. As a result, appropriate feature selection is the most important step of designing the SER system. Acoustic, linguistic, and context information are three main categories of features used in the SER research [6]. In addition to those features, hand-engineered features including pitch, Zero-Crossing Rate (ZCR), and MFCC are widely used in many research works [6,7,8,9]. More recently, convolutional neural network (CNN) has been in use at a dramatically increasing rate to address the SER problem [2,10,11,12,13].

Since the results from deep learning methods are more promising [8,14,15], we used a 3D CNN model to predict the emotion embedded in a speech signal. One challenge in SER using multi-dimensional CNNs is the dimension of speech signal. Since the purpose of this study is to learn spectra-temporal features using a 3D CNN, one must transform the one-dimensional audio signal to an appropriate representation to be able to use it with 3D CNN. A spectrogram is a 2D visual representation of short-time Fourier transform (STFT) where the horizontal axis is the time, and the vertical axis is the frequency of signal [16]. In the proposed framework, audio data is converted into consecutive 2D spectrograms in time. The 3D CNN is especially selected because it captures not only the spectral information but also the temporal information.

To train our 3D CNN using spectrograms, firstly, we divide each audio signal to shorter overlapping frames of equal length. Next, we extract an 88-dimensional vector of commonly known audio features for each of the corresponding frames. This means, at the end of this step, each speech signal is represented by a matrix of size n×88 where *n* is the total number of frames for one audio signal and 88 is the number of features extracted for each frame. In parallel, the spectrogram of each frame is generated by applying STFT. In the next step, we apply k-means clustering on the extracted features of all frames of each audio signal to select *k* most discriminant frames, namely keyframes. This way, we summarize a speech signal with *k* keyframes. Then, the corresponding spectrograms of the keyframes are encapsulated in a tensor of size k×P×Q where *P* and *Q* are horizontal and vertical dimensions of the spectrograms. These tensors are used as the input samples to train and test a 3D CNN using 10-fold cross-validation approach. Each of 3D tensors is associated with the corresponding label of the original speech signal. The proposed 3D CNN model consists of two convolutional layers and a fully connected layer which extracts the discriminative spectra-temporal features of so-called tensors of spectrograms and outputs a class label for each speech signal. The experiments are performed on three different datasets, namely Ryerson Multimedia Laboratory (RML) [17] database, Surrey Audio-Visual Expressed Emotion (SAVEE) database [18] and eNTERFACE’05 Audio-Visual Emotion Database [19]. We achieved recognition rate of 81.05%, 77.00% and 72.33% for SAVEE, RML and eNTERFACE’05 databases, respectively. These results improved the state-of-the-art results in the literature up to 4%, 10% and 6% for these datasets, respectively. In addition, the 3D CNN is trained using all spectrograms of each audio file. As a second series of experiments, we used a pre-trained 2D CNN model, say VGG-16 [20] and performed transfer learning on the top layers. The results obtained from our proposed method is superior than the ones achieved from training VGG-16. This is mainly due to fewer parameters used in the freshly trained 3D CNN architecture. Also, VGG-16 is a 2D model and it cannot detect the temporal information of given spectrograms.

The main contributions of the current work are: (a) division of an audio signal to *n* frames of equal length and selecting the *k* most discriminant frames (keyframes) using k-means clustering algorithm where k<<n; (b) representing each audio signal by a 3D tensor of size k×P×Q where *k* is the number of consecutive spectrograms corresponding to keyframes and *P* and *Q* are horizontal and vertical dimensions of each spectrogram; (c) Improving the ER rate for three benchmark datasets by learning spectra-temporal features of audio signal using a 3D CNN and 3D tensor inputs.

The main motivation of the proposed work is to employ 3D CNNs, which is capable of learning spectra-temporal information of audio signals. We proposed to use a subset of spectrogram frames which minimizes redundancy and maximizes the discrimination capability of the represented audio signal. The selection of such a subset provides a computationally cheaper tensor processing for comparable or improved performance.

The rest of the paper is organized as follows: In Section 2, we review the related works and describe steps of our proposed method. In Section 3, our experimental results are illustrated and compared with the state of the art in the literature. Finally, in Section 4 conclusion and future work is discussed.

## 2. Materials and Methods

Generally speaking, a SER system is composed of two parts: a preprocessing part that extracts suitable features and a classifier that employs those features to perform ER. This section overviews existing strategies in the SER research area [21,22].

### 2.1. Related Works

In a very recent work, ref. [23] proposed a robust technique of SER by embedding phoneme sequences and spectrograms. The authors represented each phoneme as an embedding numeric vector. They use two CNN models, a phoneme-based CNN model and a 2D CNN model for spectrograms. Both models have four parallel convolutional kernels. They used the Interactive Emotional Dyadic Motion Capture (IEMOCAP) database [24] and they achieved an overall accuracy of 73.9% on this corpus. Considering the high computational cost of training CNNs, the drawback of this method is employing two separate CNN models. Also, comparison with other benchmark databases is ignored.

In another recent work, Zhang et al. [25] achieved 70.4% accuracy on the same corpus, IEMOCAP. They proposed an attention-based fully convolutional neural network (FCN). FCNs can handle spectrograms with variable sizes. In fact, they turn AlexNet [26] into an FCN by removing its fully connected layers and then using it as an encoder. Later, they attach an attention layer which is followed by a SoftMax layer. They compared their results with a fine-tuned version of AlexNet and VGG-16 [20]. They reported 67.9% and 66.8% accuracy on IEMOCAP database. Also, they reported recognition rate of 66.5% and 65.3% by direct training (without fine tuning) of these two deep networks. The advantage of this work is that the preprocessing step is limited to the generation of so-called spectrograms.

Avots et al. [9] conducted a cross-corpus evaluation. They analyzed a model on the audio-visual information of SAVEE, RML and eNTERFACE’05 databases and tested the same model on AFEW database to merely show how challenging the task of recognizing emotional states in real world environment might be. They represented the emotional speech in SAVEE, RML and eNTERFACE’05 databases by a 1×650, 1×1725 and 1×1570 feature vector, respectively. Mainly, they used spectral features such as energy entropy, ZCR, and harmonic product spectrum to represent each audio signal. Then, they applied SVM classifier and achieved 77.4%, 69.3% and 50.2% for SAVEE, RML and eNTERFACE’05 databases and only 27.1% for AFEW database. One disadvantage of this work is the different feature vector size that is used for each dataset which ignores the generalization aspect of machine learning methods and makes it highly susceptible to overfitting on a specific dataset.

Torfi et al. [8] proposed a 3D CNN for cross audio-visual matching recognition. Their audio-visual recognition system couples two non-identical 3D CNN architecture. This can map a pair of speech and video input into a new representation space for evaluation of correspondence between them. The input that they used were spectrograms, as well as the first and second order derivatives of the MFEC features. They applied feature-level fusion of audio and video features and reported the area under the curve 95.4% for Lip Reading in the Wild dataset.

Badshah et al. [22] used spectrograms of a speech signal as the input for a 2D CNN. They extracted spectrograms of each speech signal and then split the spectrogram into several smaller spectrograms. These smaller spectrograms are later resized and used as the input to a 2D CNN architecture. They reported using rectangular shaped kernels for convolution layers help to capture local features effectively. They trained and evaluated their model on Berlin Emotional Database (EmoDB) [27] and obtained a weighted (overall) accuracy of 72.21%. Also, in [14], they reported that a freshly trained CNN performs better than transfer learning on AlexNet [26] for SER purpose.

Ref. [28] evaluated two types of neural networks: CNNs and long short-term memory networks. They used IEMOCAP corpus for training and evaluation. In the preprocessing step, they split each sentence longer than 3 s to shorter sub-sentences. The emotional label of the original sentence is assigned to sub-sentences. Then they calculate a spectrogram for each sub-sentence. They studied the effect of 10 Hz and 20 Hz grid resolution and they report using lower resolution yields lower accuracy. They obtained weighted accuracy of 68.8%. They also, used harmonic modeling to remove noise from spectrograms. We believe k-means clustering will select the frames which are less redundant and therefore the corresponding spectrogram of the selected frames is more informative.

Noroozi et al. [29] proposed an audio-visual ER system for video clips. They extracted 88 features including MFCC, pitch, intensity, mean, variance, etc. from the whole speech signal. No framing is performed. Then, they applied SVM and Random Forest on this feature space. They reported the weighted accuracy of 56.07% and 65.28% and 47.11% for SAVEE, RML and eNTERFACE’05 datasets using Random Forest. Results obtained by SVM were lower than the Random Forest. In another work from same author [6], they used random forests and decision trees to classify speech signals using a vector of size 14 para-linguistic features. They obtained an overall accuracy of 66.28% on SAVEE dataset.

Schluter and Grill [13] applied pitch-shifting and time-stretching as two significant methods for data augmentation of spectrograms. They used the augmented data as input to 2D CNN. One disadvantage of this work is that due to a huge number of spectrograms, they used a fixed number of weight updates which means the convergence of CNN optimizer is not guaranteed. Other researchers such as Palaz et al. [12] split a raw input signal to a sequence of frames, and report a class-base score for each frame by passing through several convolution filter stages and a multi-layer perceptron classifier.

CNN is used to learn affect-salient features for SER in the precious work of [7]. In the first step of training, the unlabeled samples are used to learn Local Invariant Features (LIF) using a sparse auto-encoder. In the second step, LIF is used as the input to a feature extractor. The weighted accuracy on SAVEE, EmoDB was 71.8% and 57.2%.

Abdel-Hamid et al. [15] proposed a limited-weight-sharing scheme that models the speech features for speech recognition systems while [11] proposed a new method for modeling speech signals using Restricted Boltzmann Machine.

### 2.2. Proposed Method

#### 2.2.1. Preprocessing

**Figure 1:**
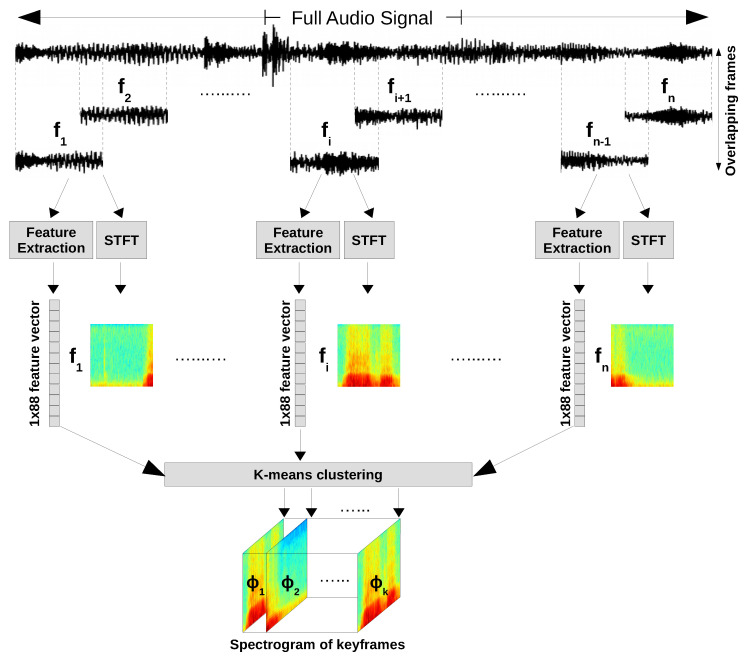
The proposed framework for preprocessing the data.

In this study, RML, SAVEE and eNTERFACE’05 datasets are used. The preprocessing pipeline is shown in Figure 1. First, the speech signals are extracted from video clips using the FFmpeg framework. Then, each speech signal is divided to shorter overlapping frames of equal length. Each frame has 50% overlap with the previous one. This step results to division of each speech signal to *n* frames. Depending on the length of speech signal, the length of frames differs from one audio signal to another, but all frames of one audio signal has the same length. Then, for each frame 88 commonly known audio features such as MFCC, pitch, variance, intensity, and filter-bank energies are extracted. We adopted the set of extracted features from [29]. The complete list of extracted features is shown in Table 1.

In parallel, the spectrogram of each frame is generated. A Spectrogram is simply a signal strength versus time at different frequencies and is generated by applying STFT. A sequence of overlapping Hamming windows is applied to each frame with window size of 20 ms [30], a window shift of 10 ms and hope size of 256. At the end of this step, each speech signal is represented by a matrix of size n×88 and *n* spectrograms as shown in Figure 1. *n* is the number of frames and matching to each frame there exist a spectrogram, i.e., each audio frame has one feature vector and a corresponding spectrogram.

In the next step, k-means clustering algorithm is applied on all extracted feature vectors of one speech signal to select *k* most discriminant frames known as keyframes. As we mentioned before, corresponding to each of these keyframes, there exist a spectrogram. The sequence of *k* successive spectrograms of the keyframes for one speech signal forms a 3D tensor representing that speech signal. Such tensors are used as the input samples for training our 3D CNN architecture. Label of the original speech signal is assigned to the generated 3D tensor. To find the best representative *k*, we started with *k* is equal to 9 and we increased it in a heuristic fashion to 18 and 27. The best *k* which maximized the accuracy over the validation set and during training is equal to 9.

Training CNNs and especially 3D CNNs is an exhaustive and time-consuming process. As a result, summarizing the input samples (a speech signal represented by a selected sequence of spectrograms) without degrading the performance becomes highly important. For example, in [13], huge number of spectrograms is produced using hop size equal to 1. Due to high redundancy of overlapping audio frames and memory limitation, training of the CNN is performed for a fixed number of 40,000 weight updates instead of training over a full dataset. This means that not only the optimizer might not converge but also, not all the spectrograms of one audio signal is observed during training. In addition, a 3D CNN can be trained as deep as possible subject to the machine memory limit and computation affordability [31]. Thus, it is desired to handle memory limitation and reducing the computational time by summarizing input samples while preserving the performance.

**Figure 2:**
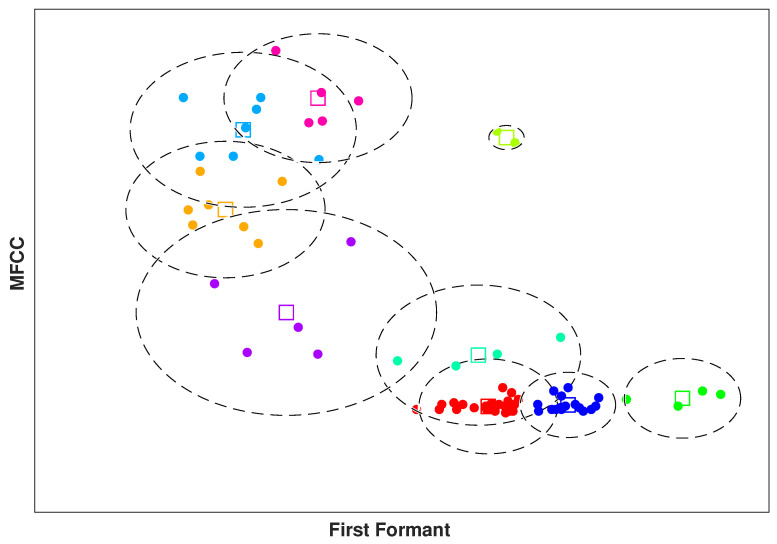
k-Means Clustering visualization for one audio sample in Angry category.

In our methodology, k-means clustering algorithm addresses these problems. Because it detects the redundancy by clustering the feature vectors representing the frames of one audio signal and maximizing the distinctions between those frames. Figure 2 shows the generated clusters and their corresponding centroids. To visualize the discrimination of clusters, we applied *t*-test score on the 1×88 feature vectors of selected frames and non-selected frames of a single audio file to find the two best representative features. The *t*-test examines the differences of two populations using the mean and standard deviation of each population. The first formant and the MFCC provided the maximum difference. The k-means clustering is visualized using the selected features by *t*-test. In the following context, first we explain feature extraction and spectrogram generation in more details. Then, the proposed 3D CNN for SER is described.

1. Extracted Features:
  Emotions can be represented using different features of speech. For example, a speaker who is angry has a faster speech rate as well as higher energy and pitch frequency. Some of the most effective features of speech for ER are duration, intonation, pitch and intensity, filter-bank energies, MFCCs, ΔMFCCs, and ZCR. In this paper, we extracted 88 features proposed by [29]. The complete list of features is shown in Table 1 and for a speech signal *s* with length *N*, they are explained in detail in Appendix A.
2. Spectrograms:
  As we mentioned before, one challenge in SER using CNNs is the dimension of speech signal. Since the purpose of this study is to learn spectra-temporal features using a 3D CNN, one must transform the one-dimensional audio signal to an appropriate representation for CNNs. One such representation is spectrogram which is the visual representation of signal strength over time at different frequencies [22]. Spectrogram is generated by applying STFT. STFT is a Fourier-based transform which determines the sinusoidal frequency and phase of local portions of a signal as it changes over time. In practice, to compute STFT, first a long time signal must be divided to shorter frames or segments of equal length. Then, by applying Fourier transform on each shorter frame, Fourier spectrum of that frame reveals. Visualizing the changing spectra as a function of time results in spectrogram [16].
  In other word, the spectrogram is a visual representation of STFT where the horizontal axis represents the time and the vertical axis represents the frequency of signal in that short frame. In a spectrogram, at a particular time point and a particular frequency, dark colors illustrate the frequency in a low magnitude, whereas light colors show the frequency in higher magnitudes. Spectrograms are perfectly suitable for variety of speech analysis including SER [16]. In this work, we aim to represent each speech signal as a selected sequence of spectrograms generated by applying STFT on overlapping frames.
3. k-means clustering:
  It is an iterative, data-partitioning algorithm that assigns each sample point to exactly one of the k clusters. First, k observations are selected randomly to be the centroids of clusters. Then the distance between each sample point and the cluster-centroids are calculated. The sample point is assigned to the cluster with the closest centroid. When all sample points are assigned to exactly one of the clusters, the average of the sample points in each cluster is computed to obtain k new centroid locations. The distance calculation step and modifying the centroid location is then repeated until clusters stabilize or a maximum number of iterations is reached [32,33].

#### 2.2.2. 3D CNN Architecture

**Figure 3:**
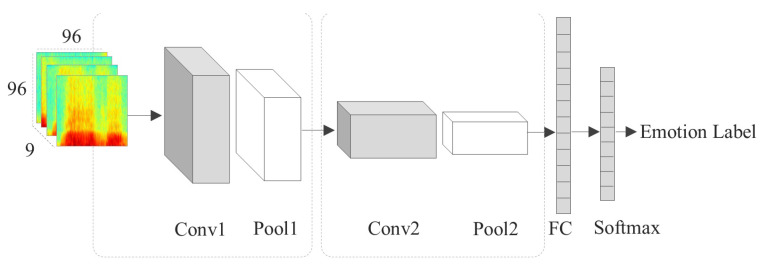
Block diagram of the proposed architecture for SER.

The proposed architecture is a 3D CNN trained using 3D tensors. Each of these tensors contain a sequence of spectrograms for one audio signal. The proposed 3D model consists of two convolutional layers, one fully connected layer, a dropout, and a SoftMax layer. In Table 2 the spatial size of the 3D kernels is reported as *T* × *H* × *W* where *T* is the kernel size in temporal dimension, and *H* and *W* are the kernel sizes in height and width dimensions, respectively. By applying a 3D kernel, spectra-temporal features are extracted using a 3D convolutional operation. The complete block diagram of our proposed architecture is shown in Figure 3. We did not use any zero padding because it adds extra zero-energy coefficients which is not meaningful in local feature extraction.

As we mentioned before, the best *k* obtained equal to 9. As a result, each input sample of our proposed network is 9 consecutive spectrograms representing one emotional speech signal. All the spectrograms obtained from the pipeline explained in Section 2.2.1 are resized to 96×96 images. The first convolution layer, Conv1 has 128 kernels of size 3×5×1 which are applied at strides of 1 pixel. The 3D convolutional layers extract the correlation between high-level temporal features and the spatial features of spectrograms. Conv1 uses a Parametric Rectified Linear Unit (PReLU). Following, a 3D max pooling layer with a kernel size 2×2×2 (Pool1) and stride 1×2×1 is used. PReLU is an activation function that is used instead of regular sigmoid ones with the aim of improving efficiency of the training process. Layer Conv2 has 256 kernels of size 3×7×1 again with a moving stride of 1. Conv2 also uses PReLU as activation function. Pool2 is a 3D max pooling layer with the same kernel size and stride as Pool1. Pool2 is followed by a dropout layer with a dropout rate of 75% to avoid overfitting. Then, one fully connected (FC) layer with 64 units and a classification layer with 6 output class is used. Also, batch normalization [34] has been used to improve the training convergence.

In the proposed 3D model, we followed best experimental observations reported in [22,31,35]. In [14], it is reported that using rectangular kernels with large heights captures the local features effectively. As a result, we used a rectangular kernel of size 3×5×1 and 3×7×1 in the convolution layers. Also, [35] reported that using shallow temporal and moderately deep spectral kernels are optimal for the SER purpose. Thus, we employed 128 and 256 filters for convolutional layers which resulted in the best performance on the validation set. Using more than 256 filters did not help to improve the performance on the validation set. For initialization of weights and bias parameters, two methods including variance scaling [8] and random uniform distributions are tested. Initialization of both parameters with random uniform distribution resulted in a better performance on the validation set. For regularization, we used *l2* weight regularization with setting the regularization factor to $5\times 10^{-4}$.

## 3. Results and Discussion

Taking into account the acquisition source of the data, three general groups of emotional databases exist: spontaneous emotions, acted emotions based on invocation and simulated emotions. Sample databases recorded in natural situations such as TV shows or movies are categorized under the first group. Usually, such databases suffer from low quality due to different sources of interference. For databases under second group, an emotional state is induced using various methods such as watching emotional video clips or reading emotional context. Although psychologists prefer this type of databases, the resulted reaction to the same stimulant may differ. Also, ethically provoking strong emotions might be harmful for the subject. eNTERFACE’05 and RML are examples of this group. The last group of databases are simulated emotions with high quality recordings and still emotional state. SAVEE database is a good example of this group.

### 3.1. Dataset

Three benchmark datasets were used to conduct the experiments, namely RML, SAVEE and eNTERFACE’05. All three datasets support audio-visual modals. Several reasons have been considered while choosing the datasets. We selected databases in a way covering a variation of size to show the flexibility of our model. Firstly, all three datasets are represented for same emotional states which makes them highly comparable. It is known that distinction between two emotion categories (for example disgust and happy) with large inter-class differences is easier than two emotions with small inter-class discrepancy. In addition, having the same number of emotional states prevents misinterpretation of the experimental results. Because as the number of emotional states increase the classification task becomes more challenging.

Second, since all three datasets recorded for both the audio and the visual modals, the quality of the recorded audios is almost the same (16-bit single channel format). For example, comparing databases recorded with high acoustic quality and for the specific purpose of SER (EmoDB) with databases recorded in real environments is not preferable. Extraction of speech signals from videos for all three datasets is performed using the FFmpeg framework. Third, SAVEE, RML and eNTERFACE’05 can be categorized as small-size, mid-size, and large-size databases. Thus, the proposed model is evaluated to have a stable performance in terms of number of input samples.

The data processing pipeline explained in Section 2.2.1 is applied on each audio sample. To avoid overfitting, in all experiments, we divided the data such that 90% is used for training and 10% for test. We performed 10-fold cross-validation on the train part which means 90% of the train data is used for training and 10% for validation. Finally, the cross validated model is evaluated on the test part. The experiments are all performed for speaker-independent scenarios.

#### 3.1.1. SAVEE

The SAVEE database has 4 male subjects who acted emotional videos for six basic emotions namely anger, disgust, fear, happiness, sadness, and surprise. A neutral category is recorded as well but since the other two datasets does not include neutral, we discard it. This dataset consists of 60 videos per category. 360 emotional audio samples extracted from the videos of this dataset.

#### 3.1.2. RML

The RML database represented by Ryerson Multimedia Laboratory [17] includes 120 videos in each of six basic categories mentioned above from 8 subjects spoke various languages such as English, Mandarin, and Persian. A dataset of 720 emotional audio samples is obtained from this database.

#### 3.1.3. eNTERFACE’05

The third dataset is eNTERFACE’05 [19] recorded from 42 subjects. All the participants spoke English and 81% of them are female. Each subject was asked to perform all six basic emotional states. Emotional states are exactly the same as SAVEE and RML. 210 audio samples per category is extracted from this dataset.

### 3.2. Experiments

To assess the proposed method, four experiments are conducted on each dataset. In the first experiment, we trained the proposed 3D CNN model using the spectrograms of selected keyframes by applying 10-fold cross-validation method. In the second experiment 3D CNN model is trained using spectrograms of all frames. In the third experiment, by means of transfer learning, we trained VGG-16 [20] using the spectrograms of keyframes. Finally, in the last experiment we trained VGG-16 using all spectrograms generated for each audio signal. Comparing the results obtained from the second and third experiment shows that k-means clustering discarded the audio frames which convey insignificant or redundant information. This can be interpreted from the results given in Table 3, Table 4 and Table 5 which does not differ notably. It is important to note that the overall accuracy results obtained from these four experiments are shown by Proposed 3D CNN${}^{\left(1\right)}$, Proposed 3D CNN${}^{\left(2\right)}$, VGG-16${}^{\left(1\right)}$ and VGG-16${}^{\left(2\right)}$ in those tables.

#### 3.2.1. Training the Proposed 3D CNN

The CNN architecture illustrated in Figure 3 was trained on a sequence of 9 consecutive spectrograms paired with the emotional label of the original speech sample. We train the network for 400 epochs with assuring that each input sample consists of a sequence of 9 successive spectrograms. Also, as a second experiment, the proposed 3D CNN was trained using all spectrograms of each audio signal.

**Figure 4:**
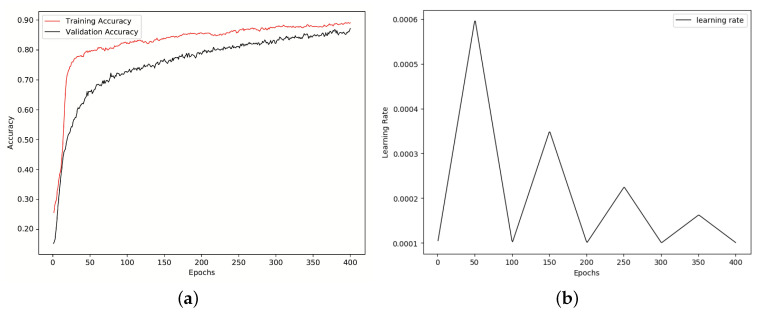
Results on RML database. (**a**) Training versus validation, accuracy improvement; (**b**) Cyclical learning rate decay.

Updates are performed using Adam optimizer [37], categorical cross-entropy error, mini-batches of size 32 [13] and a triangular cyclical learning rate policy by setting the initial learning rate to $1\times 10^{-4}$, maximum learning rate to $6\times 10^{-4}$, cycle length to 100 and step size to 50. Cycle length is the number of iterations until the learning rate returns to the initial value [38]. Step size is set to half of the cycle length. Figure 4b shows the learning rate for 400 iterations on RML dataset. As we mentioned before, to fight overfitting, we used *l2* weight regularization with factor $5\times 10^{-4}$. In all experiments, 90% of the data is used for training and the rest for test. This means, the model learned spectra-temporal features by applying 10-fold cross-validation on the training part of the data. Then, the trained model is evaluated using the test data.

The average accuracy on test set of SAVEE, RML and eNTERFACE’05 databases is illustrated as a confusion matrix in Table 6, Table 7 and Table 8, respectively. Clearly, the proposed method achieved superior results than the state-of-the-arts in the literature. Since the complexity of CNNs are extremely large, using discriminant input samples is of high importance especially when it comes to real-time applications. To the best of our knowledge, this is the first paper representing a whole audio signal by means of *k* most discriminant spectrograms. This means, speech signal can be represented with fewer frames, yet preserving the accuracy. Figure 4a shows the training and validation accuracy improvement for RML dataset over 400 iterations. Also, Figure 4b shows the cyclical learning rate decay over same number of iterations and same dataset.

#### 3.2.2. Transfer Learning of VGG-16

In the next two experiments, we selected one of the well-known 2D CNNs, VGG-16 [20]. We applied transfer learning on the top layers to make it more suitable for the SER purpose. We trained the network for 400 weight updates. The initial learning rate is set to $1\times 10^{-4}$.

In the first scenario, only the selected spectrograms of audio signals are given to VGG-16. In the second scenario, without applying k-means clustering algorithm, all generated spectrograms for each audio signal are used. In both cases, majority voting is used to make a final decision for each audio signal and assign a label to it. This means majority of labels predicted for the spectrograms of one audio is considered to be the final label for that audio signal. Both experiments under-performed the proposed 3D CNN.

This is mainly because VGG-16 is pre-trained on ImageNet dataset [39] for object detection and image classification purposes. Also, it has more complexity to adjust its weight. As a result, transfer learning was not helpful. Same conclusion has been reported by [14] and [25] for applying transfer learning on AlexNet using spectrograms. Fewer parameters in the freshly trained 3D CNN is the main reason for achieving the higher performance. The overall accuracy obtained by these experiments is compared with the state of the art in the literature in Table 3, Table 4 and Table 5 for SAVEE, RML and eNTERFACE’05 datasets, respectively.

## 4. Conclusions

In this paper, we studied the performance of 3D Convolutional Neural Networks using spectrograms. Instead of using the whole set of spectrograms corresponding to the audio frames, we selected *k* best frames for representing the whole speech signal. We compared the results of the proposed 3D CNN with the results obtained from 2D CNNs. It shows that the proposed method performs better than the pre-trained 2D networks. Future works may include comparing with pre-trained 3D-architecture such as Res-3D and C3D or applying different types of data augmentation to improve the results by fighting the overfitting. Fusion with visual data is another direction to study the multimodal performance of 3D architectures as well as cross-correlation between different modalities.

## Figures and Tables

**Table 1:** List of extracted features for each audio frame.

Feature	#	Feature	#	Feature	#
Intensity	1	Pitch	1	Median	1
Standard Deviation	1	Mean	1	Harmonic mean	1
Minimum amplitude	1	maximum amplitude	1	Percentile	1
Zero-Crossing Rate (ZCR)	1	ΔMFCCs	13	MFCCs	13
ZCR Density	1	Formants	20	Autocorrelation	1
Filter-Bank Energies	26	Formant bandwidth	4		
**Total**					**88**

**Table 2:** The resolution of the proposed 3D CNN.

Layer	Input-Size	Output-Size	Kernel	Stride
Conv1	9×96×96×3	7×92×96×128	3×5×1	1
Pool1	7×92×96×128	6×46×95×128	2×2×2	1×2×1
Conv2	6×46×95×128	4×40×95×256	3×7×1	1
Pool2	4×40×95×256	3×20×94×256	2×2×2	1×2×1
dropout	3×20×94×256	3×20×94×256	-	-
FC	1,443,840	64	-	
Dense	64	6	-	
Dense	6	-	-	

**Table 3:** Comparison of recognition rates among different methods for SAVEE dataset.

Method	Audio Representation	Accuracy
SVM [9]	feature vector ^†^	77.4%
SVM [29]	feature vector ^†^	48.81%
Random Forest [29]	feature vector ^†^	56.07%
2D CNN [7]	Spectrograms	73.6%
VGG-16${}^{\left(1\right)}$	all spectrograms ^*^	49.20%
VGG-16${}^{\left(2\right)}$	*k* selected spectrograms ^⋄^	45.11%
Proposed 3D CNN${}^{\left(1\right)}$	all spectrograms ^*^	80.41%
Proposed 3D CNN${}^{\left(2\right)}$	*k* selected spectrograms ^⋄^	81.05%

^†^: A feature vector of commonly known audio features like Table 1. ^*^: All generated frames/spectrograms of one audio is used. ^⋄^: Only *k* (9) frames/spectrograms of one audio is used.

**Table 4:** Comparison of recognition rates among different methods for RML dataset.

Method	Audio Representation	Accuracy
SVM [9]	feature vector ^†^	69.30%
SVM [29]	feature vector ^†^	43.47%
Random Forest [29]	feature vector ^†^	65.28%
VGG-16${}^{\left(1\right)}$	all spectrograms ^*^	43.58%
VGG-16${}^{\left(2\right)}$	*k* selected spectrograms ^⋄^	41.17%
Proposed 3D CNN${}^{\left(1\right)}$	all spectrograms ^*^	71.44%
Proposed 3D CNN${}^{\left(2\right)}$	*k* selected spectrograms ^⋄^	77.00%

^†^: A feature vector of commonly known audio features like Table 1. ^*^: All generated frames/spectrograms of one audio is used. ^⋄^: Only *k* (9) frames/spectrograms of one audio is used.

**Table 5:** Comparison of recognition rates among different methods for eNTERFACE05 dataset.

Method	Audio Representation	Accuracy
SVM [9]	feature vector ^†^	50.2%
SVM [29]	feature vector ^†^	41.32%
Random Forest [29]	feature vector ^†^	47.11%
HMM [36]	feature vector ^†^	52.19%
VGG-16${}^{\left(1\right)}$	all spectrograms ^*^	33.33%
VGG-16${}^{\left(2\right)}$	*k* selected spectrograms ^⋄^	39.23%
Proposed 3D CNN${}^{\left(1\right)}$	all spectrograms ^*^	69.50%
Proposed 3D CNN${}^{\left(2\right)}$	*k* selected spectrograms ^⋄^	72.33%

^†^: A feature vector of commonly known audio features like Table 1. ^*^: All generated frames/spectrograms of one audio is used. ^⋄^: Only *k* (9) frames/spectrograms of one audio is used.

**Table 6:** Confusion matrix for SAVEE.

	Ang	Dis	Fea	Hap	Sad	Sur
Ang	0.89	0.02	0	0	0	0.08
Dis	0	0.9	0.1	0	0	0
Fea	0	0	0.75	0.08	0.17	0
Hap	0	0.08	0	0.75	0.08	0.08
Sad	0.08	0.1	0	0	0.81	0
Sur	0.08	0.08	0.08	0	0	0.76
**Average RR%**						81.05

**Table 7:** Confusion matrix for RML.

	Ang	Dis	Fea	Hap	Sad	Sur
Ang	0.92	0.08	0	0	0	0
Dis	0	0.67	0.25	0.08	0	0
Fea	0	0.17	0.7	0.04	0	0.08
Hap	0.08	0	0.08	0.75	0.08	0
Sad	0	0.08	0.08	0	0.83	0
Sur	0.08	0.17	0	0	0	0.75
**Average RR%**						77.00

**Table 8:** Confusion matrix for eNTERFACE’05.

	Ang	Dis	Fea	Hap	Sad	Sur
Ang	0.92	0.08	0	0	0	0
Dis	0	0.67	0.25	0.08	0	0
Fea	0	0.17	0.42	0.08	0.25	0.08
Hap	0.08	0	0.08	0.75	0.08	0
Sad	0	0.08	0.08	0	0.83	0
Sur	0.08	0.17	0	0	0	0.75
**Average RR%**						72.33

## Abbreviations

The following abbreviations are used in this manuscript:

HCI	Human–Computer Interaction
SER	Speech Emotion Recognition
ZCR	Zero-Crossing Rate
MFCC	Mel Frequency Cepstral Coefficient
CNN	Convolutional Neural Network
STFT	Short-Term Fourier Transform
IEMOCAP	Interactive Emotional Dyadic Motion Capture
FCN	Fully Convolutional Neural Network
SVM	Support Vector Machine
RML	Ryerson Multimedia Laboratory database
LIF	Local Invariant Features
SAVEE	Surrey Audio-Visual Expressed Emotion database
HMM	Hidden Markov Model

## Appendix A

In this section, we explain the extracted features given in Table 1 with more detail.

### Appendix A.1.

1. The loudness of speech signal or the syllable peak is perceived as intensity. In another word, intensity is the power conveyed by speech signal per unit area in a direction perpendicular to that area. It can be expressed as follows:
  (A1) $$I\left(dB\right)=10\log_{10}\left[\frac{I}{I_{0}}\right]$$
  where *I* is the intensity and $I_{0}$ is the standard threshold of hearing intensity at 1000 Hz for the human ear which represented in terms of sound intensity by a value equal to $10^{\left(-12\right)}$ watts/m${}^{2}$ [40].
2. Pitch is known as the fundamental frequency of the speech signal. It can be measured either using statistical methods or in the time-frequency domains. It can be calculated as follows:
  (A2) $$\rho_{0}\left(s\right)=F\{log|F(s.w_{n}^{H}∥s∥)|\}$$
  where $w_{n}^{H}$ is the Hamming window and it is defined as follows:
  (A3) $$w_{n}^{H}=0.54-0.46\cos\left(\frac{2\pi n}{L}\right),\;\;1\leq n\leq N-1$$
  *L* is the order of the filter and it is equal to filter length −1 [29].
3. Mean of each frame is calculated as:
  (A4) $$\mu =\frac{1}{N}\sum_{i=1}^{N}s_{i}$$
4. Standard deviation is extracted by calculating the following formula:
  (A5) $$std=\sqrt{\frac{1}{N-1}\sum_{i=1}^{N}{\left(s_{i}-\mu \right)}^{2}}$$
  where μ is the mean of audio frame and $s_{i}$ shows the value of audio frame at *i*.
5. Zero-Crossing Rate (ZCR) of an audio frame is the number of times the signal passes zero or changes sign during the frame. The ZCR is expressed as below by [41]:
  (A6) $$Z\left(n\right)=\frac{1}{2}\sum_{m=1}L\left|sgn\left[\left(m+1\right)\right]-sgn\left[x\left(m\right)\right]\right|$$
  where
  (A7) $$sgn\left[x\left(m\right)\right]=\left\{\begin{array}{cc} +1 & \mathrm{if}\;x(m)\geq 0\\ -1 & \mathrm{if}\;x(m)<0 \end{array}\right.$$
  A high ZCR is indicative of a stationary series.
6. With an input signal starting at time zero and stopping at time *T*, the probability distribution satisfies [42]:
  (A8) $$P\left(g<u\right)=\frac{2}{\pi }\arcsin\sqrt{\frac{u}{T}}$$
  where *g* is the last time that the signal passed zero. The density function is then:
  (A9) $$P\left(u\right)=\frac{1}{\pi }\frac{1}{\sqrt{u(T-u)}}$$
7. Harmonic mean is computed using the following formula:
  (A10) $$\bar{m}=\frac{N}{\sum_{i=1}^{N}\frac{1}{s_{i}}}$$
8. Maximizing the inner product of the speech signal by its shifted version is another important feature that can be computed using the autocorrelation function r(τ) where τ is the time shift.
  (A11) $$r\left(\tau \right)=\frac{1}{N}\sum_{0}^{N-1}s\left(n\right)s\left(n+\tau \right)$$
9. In calculation of MFCC, the formula proposed by Davis et al. [43] is used.
  (A12) $$MFCC_{i}=\sum_{\theta =1}^{N}\cos\left[i\left(\theta -1\right)\frac{\pi }{N}\right],\;i=1,\ldots ,M\;and\;\theta =1,\ldots ,N$$
  *M* and *N* are the number of extracted cepstrum coefficients and number of band-pass filters, respectively. θ denotes the log energy of θth filter.
10. Calculation of the filter-bank energies and their derivatives are performed using a first order Finite Impulse Response (FIR). An array of band-pass filters that breaks up the input signal into multiple components is called a filter bank. Each separated component carries a single frequency sub-band of the original input signal. Let the unit-sample response impulse response $h_{n}$ be the response of a discrete-time signal to a unit-sample impulse $\delta_{n}$ where $\delta_{n}=1$ for n=0 and $\delta_{n}=0$ for n≠0. Then, for an arbitrary input signal $s_{n}$, the output $y_{n}$ is given by:
  (A13) $$y_{n}=\sum_{i=0}^{M}\alpha_{i}s\left(n-1\right)+\sum_{j=1}^{N}\beta_{j}y\left(n-j\right)$$
  $\alpha_{i}$ and $\beta_{j}$ are coefficients of FIR filter and *M* is the order of the filter function. The calculation of FBEs are as follows:
  (A14) $$y\left(m\right)=\sum_{\theta =0}^{L-1}h\left(\theta \right)s\left[\left(m-\theta \right)\;mod\left(N\right)\right],\;\;m=0,1,\ldots ,N$$
  where *L* is the length of the filter [29].
11. Also, ΔMFCC is obtained using the proposed formula by [44]
  (A15) C(n)=DCT∗log(y(m))
  where DCT is the discrete cosine transform.
